# Evolution, dynamic expression changes and regulatory characteristics of gene families involved in the glycerophosphate pathway of triglyceride synthesis in chicken (*Gallus gallus*)

**DOI:** 10.1038/s41598-019-48893-9

**Published:** 2019-09-04

**Authors:** Liyu Yang, Ziming Liu, Kepeng Ou, Taian Wang, Zhuanjian Li, Yadong Tian, Yanbin Wang, Xiangtao Kang, Hong Li, Xiaojun Liu

**Affiliations:** 1 College of Animal Science and Veterinary Medicine, Henan Agricultural University, Zhengzhou, 450002 China; 2 Academic Unit of Ophthalmology, Bristol Medical School, University of Bristol, Bristol, BS8 1TD UK; 3 Henan Innovative Engineering Research Center of Poultry Germplasm Resource, Zhengzhou, 450002 China; 4 International Joint Research Laboratory for Poultry Breeding of Henan, Zhengzhou, 450002 China

**Keywords:** Gene expression, Gene regulation

## Abstract

It is well documented that four gene families, including the glycerophosphate acyltransferases (GPATs), acylglycerophosphate acyltransferases (AGPATs), lipid phosphate phosphohydrolases (LPINs) and diacylglycerol acyltransferases (DGATs), are involved in the glycerophosphate pathway of *de novo* triglyceride (TG) biosynthesis in mammals. However, no systematic analysis has been conducted to characterize the gene families in poultry. In this study, the sequences of gene family members in the glycerophosphate pathway were obtained by screening the public databases. The phylogenetic tree, gene structures and conserved motifs of the corresponding proteins were evaluated. Dynamic expression changes of the genes at different developmental stages were analyzed by qRT-PCR. The regulatory characteristics of the genes were analyzed by *in vivo* experiments. The results showed that the *GPAT*, *AGPAT* and *LPIN* gene families have 2, 7 and 2 members, respectively, and they were classified into 2, 4 and 2 cluster respectively based on phylogenetic analysis. All of the genes except *AGPAT1* were extensively expressed in various tissues. Estrogen induction upregulated the expression of *GPAM* and *AGPAT2*, downregulated the expression of *AGPAT3*, *AGPAT9*, *LPIN1* and *LPIN2*, and had no effect on the expression of the other genes. These findings provide a valuable resource for further investigation of lipid metabolism in liver of chicken.

## Introduction

Triglyceride (TG) is the most abundant lipids in humans and animals, and TG decomposition products can be used as an energy supply in most tissues. Triglyceride is synthesized via two main pathways *in vivo*, namely, the monoacylglycerol pathway and the glycerophosphate pathway^1^. The monoacylglycerol pathway occurs in the small intestine of mammals and mainly utilizes lipid decomposition products obtained from diet as a substrate to generate TGs^2^. The glycerophosphate pathway is the main pathway for most TG synthesis, and consists of four steps that are catalyzed by the glycerophosphate acyltransferase (*GPAT*), acylglycerophosphate acyltransferase (*AGPAT*), lipid phosphate phosphohydrolase (*LPIN*) and diacylglycerol acyltransferase (*DGAT*) gene families, respectively^3^.

The *GPAT* gene family acts on the first step in the glycerophosphate pathway, catalyzing the conversion of glycerol-3-phosphate and acyl-CoA to form 1-acylglycerol-3-phosphate (lysophosphatidate, LPA). It is considered to be a rate-limiting enzyme because *GPATs* exhibit the lowest specific activity of enzymes in the pathway^3^. The *GPAT* gene family contains four identified members (*GPAT1*, *GPAT2*, *GPAT3* and *GPAT4*) in mammals. Overexpression of *GPAT1* in mouse and rat livers not only leads to increased incorporation of C16:0 fatty acids (FAs) into LPA, diacylglycerol (DAG) and TG^4^, but also causes hepatic steatosis, hyperlipidemia and other diseases^5^. Moreover, the transcription factors NF-Y and SREBP-1 increases the *GPAT1* mRNA levels during adipocyte differentiation^3^. Interestingly, the mRNA expression of *GPAT2* does not increase in the livers of rats after fasting, indicating that *GPAT2* contributes little to diet-induced liver TG synthesis^6^. The *GPAT2* activity in *GPAT1* −/− mice does not prevent decrease in TG content in the liver. In addition, starvation or heavy feeding has no effect on the expression level of the *GPAT2* gene in the liver^7^. Knockdown of *GPAM* gene remarkably reduces TG synthesis and the expression of lipid metabolism-related genes in bovine embryonic fibroblast (BEF) cells^8^.

The *AGPAT* gene family acts on the second step of the pathway, catalyzing the conversion of LPA to phosphatidic acid (PA) by adding an acyl group to the sn-2 position of the glycerol backbone via fatty acyl-CoA^7^. Ten gene members have been identified in the family so far. Studies showed that *AGPAT1* could be stably overexpressed in both 3T3-L1 adipocytes and C2C12 myotubes, suggesting that *AGPAT1* might play a role in adipose and muscle tissue^9^. Compelling evidence suggested that *AGPAT2* plays a vital role in human adipocytes. Mutation of the *AGPAT2* gene leads to congenital systemic lipodystrophy in humans^10^. Knockdown of *AGPAT2* results in decreased expression of the adipogenic proteins PPARγ and C/EBP as well as delayed induction of the mature adipocyte markers aP2 and Glut4, and decreased TG accumulation in adipocytes^11^. The *AGPAT3*, *AGPAT4* and *AGPAT5* have AGPAT activity when oleoyl-CoA is used as an acyl donor, but the activity is less than 50% of *AGPAT2*. It was indicated that compounds other than acyl-CoA or LPA may be more specific substrates for these enzymes^7,12^. The *AGPAT6* is localized in the endoplasmic reticulum (ER), which does not exhibit AGPAT activity but shows GPAT activity instead. It was therefore speculated to be the second ER-localized *GPAT* and renamed *GPAT4*^13^. Studies have suggested that *AGPAT8* only has approximately 20% of the activity of *AGPAT2*, and it can act as an acyl-CoA: lysocardiolipin acyltransferase (*ALCAT1*). *AGPAT7* is a member of the lysophosphatidylcholine acyltransferase (*LPCAT*) family and has slight AGPAT activity^14,15^. *AGPAT10*, which has identical sequence to *GPAT3*, has dual activities of AGPAT and GPAT^16,17^.

The *LPIN* gene family acts on the third step in the pathway to form DAG by dephosphorylating PA. Three members of the *LPIN* gene family have been identified in mammals. *LPIN1* is highly expressed in white and brown adipose tissue^18^, and acts as a transcriptional coactivator in the liver^19,20^. Deficiency of *LPIN1* could affect the accumulation of TG in the adipose tissue of mice^18,21^. The expression trend of *LPIN2* in adipocytes is opposite of *LPIN1*, which reaches the highest level in preadipocytes and decreases in mature adipose cells^22^. Both *LPIN2* and *LPIN3* exhibit phosphatidate phosphatase (PAP) activity. However, their activities are approximately a quarter of that of *LPIN1* produced by the 293 T cell overexpression system^23^.

The final step in the pathway is catalyzed by *DGAT1* and *DGAT2*, which acylate DAG to form TGs^24^. However, *DGAT1* and *DGAT2* belong to different gene families^25,26^. The *DGAT1* gene is a member of the acyl-CoA acyltransferase (*ACTA*) gene family^27^, which participates in the enrichment of TG, the re-esterification of fatty acid (FA) and the formation of very-low-density lipoprotein (VLDL)^28^. It was reported that, *DGAT1* mRNA levels is significantly increased during the differentiation of 3T3-L1 cells into adipocytes^27^. The *DGAT2* gene belongs to the *DGAT* gene superfamily^29,30^, which is closely related to fat deposition, backfat thickness and milk production traits^31,32^. In addition, studies have also showed that chicken sterol acyltransferase 1 (SOAT1) has DGAT1 enzyme activity, thus the chicken *SOGAT1* gene was classified into the *DGAT* gene family. However, the *DGAT1* gene has not been found in the chicken genome^33^.

Although the four gene families in the glycerophosphate pathway have been extensively studied in mammals, their evolutionary characteristics, spatial and temporal expression profiles and regulatory characteristics have rarely been reported in chicken. The liver is the major site for TG synthesis in chicken^34^. During the hen’s laying cycle, TG and other hydrophobic lipids are synthesized to meet the requirements of fast-developing oocytes^35^. The mRNA abundance of *AGPAT9* is greater in adipose tissue than in other tissues of hen^36^. The isoforms of *LPIN1* gene, *Lpin1-α* and *Lpin1-β* exhibit different tissue expression characteristics and are prominently expressed in ovary and muscle tissues, respectively^37^. A polynucleotide polymorphism g.258 M > N was found in the *LPIN1* 5′ flanking region, and both g.258 M > N and another g.65 C > T variant show a significant correlation with muscle fiber traits in chicken, suggesting a new role of *LPIN1* in muscle fiber development^37^. A recent study reported that polymorphism of the *DGAT2* gene g.13955CT was strikingly associated with birth weight and intramuscular fat (*P* < 0.05) in chicken^38^. Our previous studies identified two members of the *DGAT* gene family in chicken, *DGAT2* and *SOGAT1*, which were widely expressed in various tissues. However, their mRNA expression levels were markedly downregulated in the liver during the peak laying period and showed no change in primary hepatic cells after 17β-estradiol treatment, implying that other members rather than these genes might play a major roles in estrogen-induced TG synthesis and metabolism in chicken^33^.

While, in chicken, a systemic evaluation about the evolution, expression and regulatory characteristics of the first three gene families involved in the glycerophosphate pathway of TG biosynthesis in liver is not reported. In this study, sequences of member genes in these families were sought by screening public databases. The evolutionary origins and dynamic expression changes in members of the three gene families were explored by phylogenetic tree and qRT-PCR analyses, respectively. Furthermore, gene expression regulation was investigated by *in vivo* experiments. These findings are a valuable resource for future studies on liver lipid metabolism regulation mechanisms in chicken.

## Results

### Identification and phylogenetic analysis of *GPAT*, *AGPAT* and *LPIN* gene families in chicken

**Figure 1:**
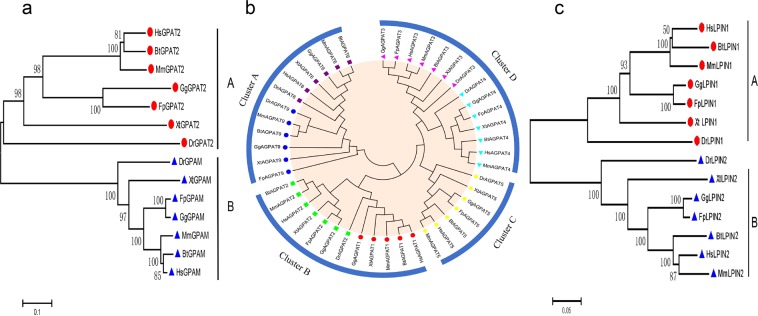
Phylogenetic analysis of the GPAT, AGPAT and LPIN in chicken. The phylogenetic tree was constructed using MEGA 6.0 software by the neighbor-joining (NJ) method, that was based on the Clustal W alignment of amino acid sequences between different species of three families. Signs of special color-shapes to distinguish the members of three gene families. (**a**–**c**) Phylogenetic relationships of GPATs, AGPATs and LPINs from chicken and other species.

By searching public databases, 2 *GPAT* genes (*GPAT2* and *GPAM*), 7 *AGPAT* genes (*AGPAT1*, *AGPAT2*, *AGPAT3*, *AGPAT4*, *AGPAT5*, *AGPAT6 and AGPAT9*) and 2 *LPIN* genes (*LPIN1 and LPIN2*) were identified in the chicken genome. Amino acid sequences of the products of these genes from diverse species, including mammals (human, mouse and cattle), avians (chicken and falcon), amphibians (tropical clawed frog) and fish (zebrafish), were retrieved from GenBank (see Supplementary Table S1a–c). Based on sequence similarity, phylogenetic tree analysis of each gene family members revealed that members of the *GPAT* and *LPIN* families were grouped into 2 clusters, which were named as cluster A and B (Fig. 1a,c), respectively. The *AGPAT* family was divided into 4 clusters, which were named as cluster A, B, C and D, indicating that these multigene families could be genetically classified into several subfamilies. Except for cluster C, the other clusters all contain 2 AGPAT members (Fig. 1b). These results clearly indicated that the orthologous genes from different species were classified into the same cluster in the phylogenetic tree and members in the multigene families might possess diverse functions.

### Analyses of the conserved motifs and gene structure of *GPAT, AGPAT* and *LPIN* gene families in chicken

**Figure 2:**
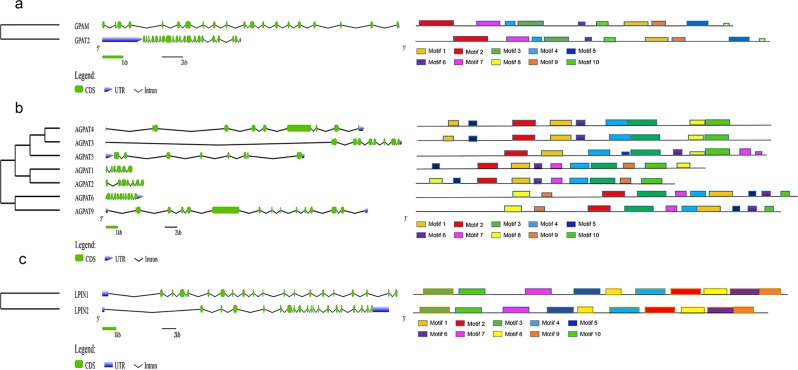
The motif and exon/intron structure analysis of three gene families in chickens. The ten color boxes represent different motifs and their positions in each gene family sequence. The introns, exons and UTR were represented by black lines, green boxes and blue wedge, respectively.

To gain insight into the evolution of the *GPAT*, *AGPAT* and *LPIN* gene families, the conserved motifs in each of family members were identified by the MEME software. In the *GPAT* gene family, both GPAT2 and GPAM harbored motifs 1–10 (Fig. 2a). For the *AGPAT* gene family, the pairs AGPAT1 and AGPAT2, AGPAT3 and AGPAT4, and AGPAT6 and AGPAT9 shared similar functional motifs (Fig. 2b), while AGPAT5 exhibited its unique motif structure. Similarly, LPIN1 and LPIN2 also shared similar functional motifs (Fig. 2c). Motif analysis results indicated that the distribution of 10 most enriched motifs was highly conserved in the same subfamily, which might correlate with gene functions.

The analysis of exon-intron organizations of each gene in the three gene families indicated that both GPAT2 and GPAM contain 20 exons in the GPAT family (Fig. 2a), AGPAT1, 2, 3, 4, 5, 6, and 9 contain 6, 8, 9, 8, 10, 10 and 13 exons, respectively, in the *AGPAT* family (Fig. 2b), and both LPIN1 and LPIN2 contain 19 exons in the *LPIN* gene family (Fig. 2c).

### Expression profiles of the *GPAT*, *AGPAT* and *LPIN* gene families in different tissues

**Figure 3:**
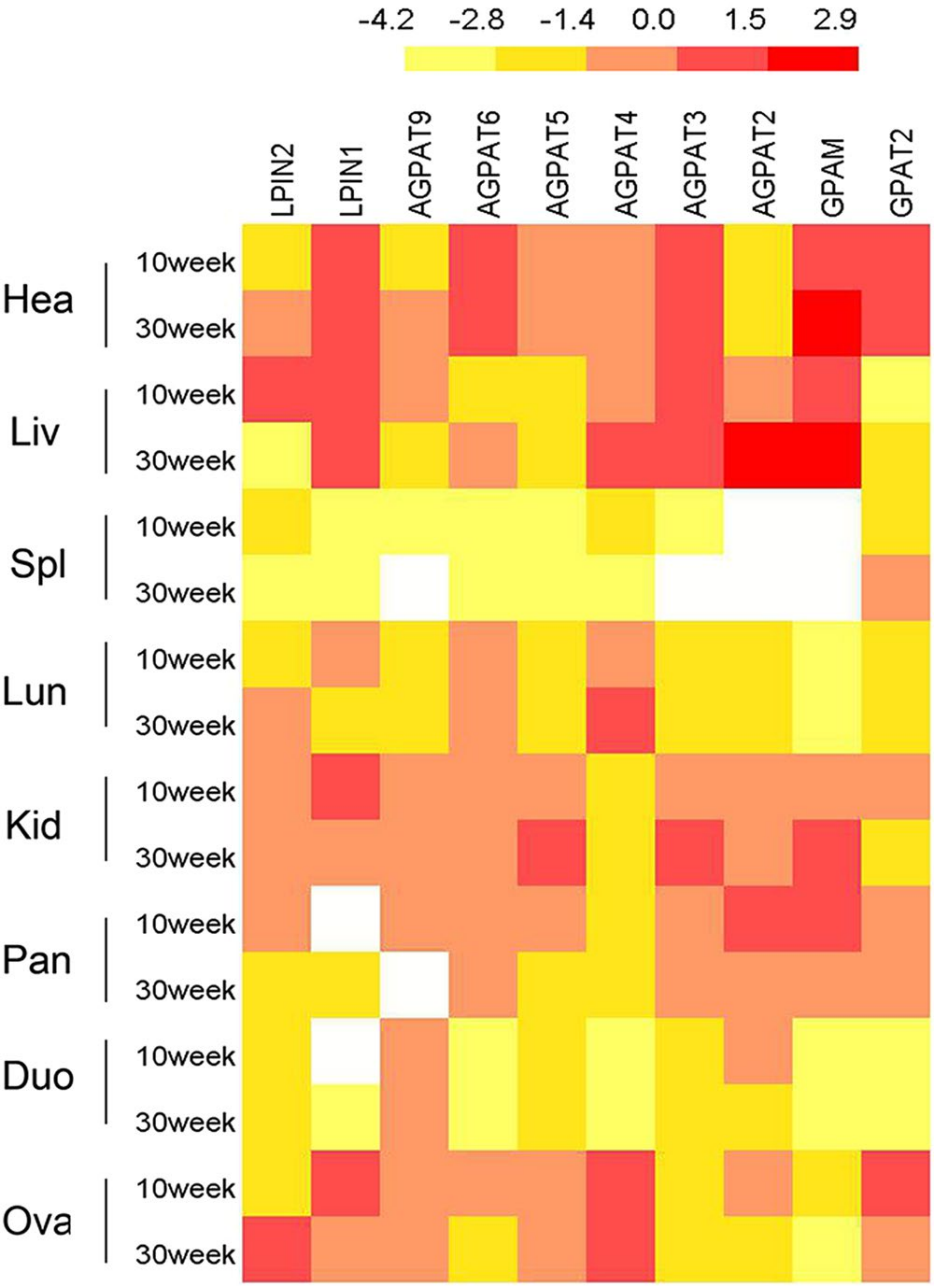
Expression profiles of three gene families in different tissues in chicken. Tissues include heart (hea), liver (liv), spleen (spl), lung (lun), kidney (kid), pancreas (pan), duodenal (duo) and ovary (ova). The heat map was created based on Log2 transformation of mRNA expression values. A color scale bar (representing average log signal values) is shown on the top. Data were presented as mean ± SD (n = 6).

The tissue distributions of the three gene families were determined by qPCR analysis. In the *GPAT* family, *GPAM* was expressed at a relatively high level in heart and liver, at a moderate level in kidney, and at low levels in spleen and duodenum, whereas *GPAT2* was expressed in heart, kidney, pancreas, and ovary at moderate levels. In the *AGPAT* family, *AGPAT2* was highly expressed in liver and duodenum, *AGPAT4* and *AGPAT6* were highly expressed in ovary and heart, respectively. It was interesting to notice that *AGPAT3*, *AGPAT5* and *AGPAT9* had similar expression patterns in all tissues with moderate or low levels. Surprisingly, *AGPAT1* was not expressed in any of the tissues tested. In the *LPIN* family, *LPIN1* and *LPIN2* were broadly expressed in all tissues. Especially, *LPIN1* was highly expressed in liver and *LPIN2* was highly expressed in ovary; both of them were moderately expressed in heart and kidney (Fig. 3).

### Expression profiles of *GPAT*, *AGPAT* and *LPIN* families in chicken livers at different development stages

**Figure 4:**
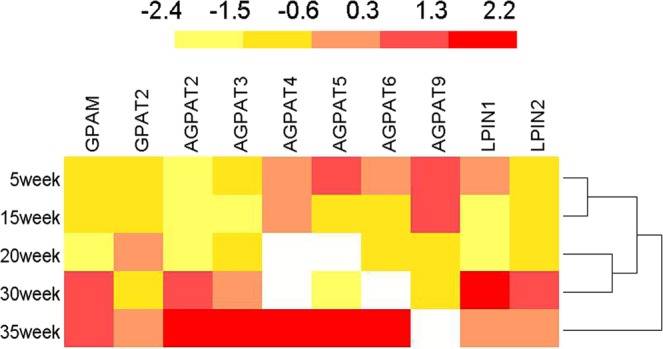
Expression profiles of three gene families in liver at different developmental stages of chicken. All data are presented as fold changes compared with gene expression in the 5 weeks group. Log2 transformed mRNA expression value was used to create the heat map. A color scale bar (representing average log signal values) was shown on the top. Data were presented as Mean ± SD (n = 6).

Liver is the major organ of lipid metabolism. To further understand the functions of the *GPAT*, *AGPAT* and *LPIN* gene families in lipid metabolism, their expression patterns in chicken livers at different developmental stages were determined. *GPAM* and *GPAT2* mRNA expression levels were lower in the early stage of growth and development (5–20 weeks old) and then increased remarkably during the peak laying period (30–35 weeks old). The expression levels of *AGPAT*2, 3, 4, 5, and 6 gradually increased with age from 5 weeks old to 35 weeks old, while *AGPAT9* showed the opposite trend, where its expression declined to the lowest level at 30 weeks old. In the *LPIN* family, the *LPIN1* and *LPIN2* mRNA expression levels were lower in the early stage of growth and development (5–20 weeks old), but increased significantly during peak-laying period (30–35 weeks old) (Fig. 4).

### Effects of 17β-estradiol on the mRNA expression of *GPAT*, *AGPAT* and *LPIN* families *in vivo*

**Figure 5:**
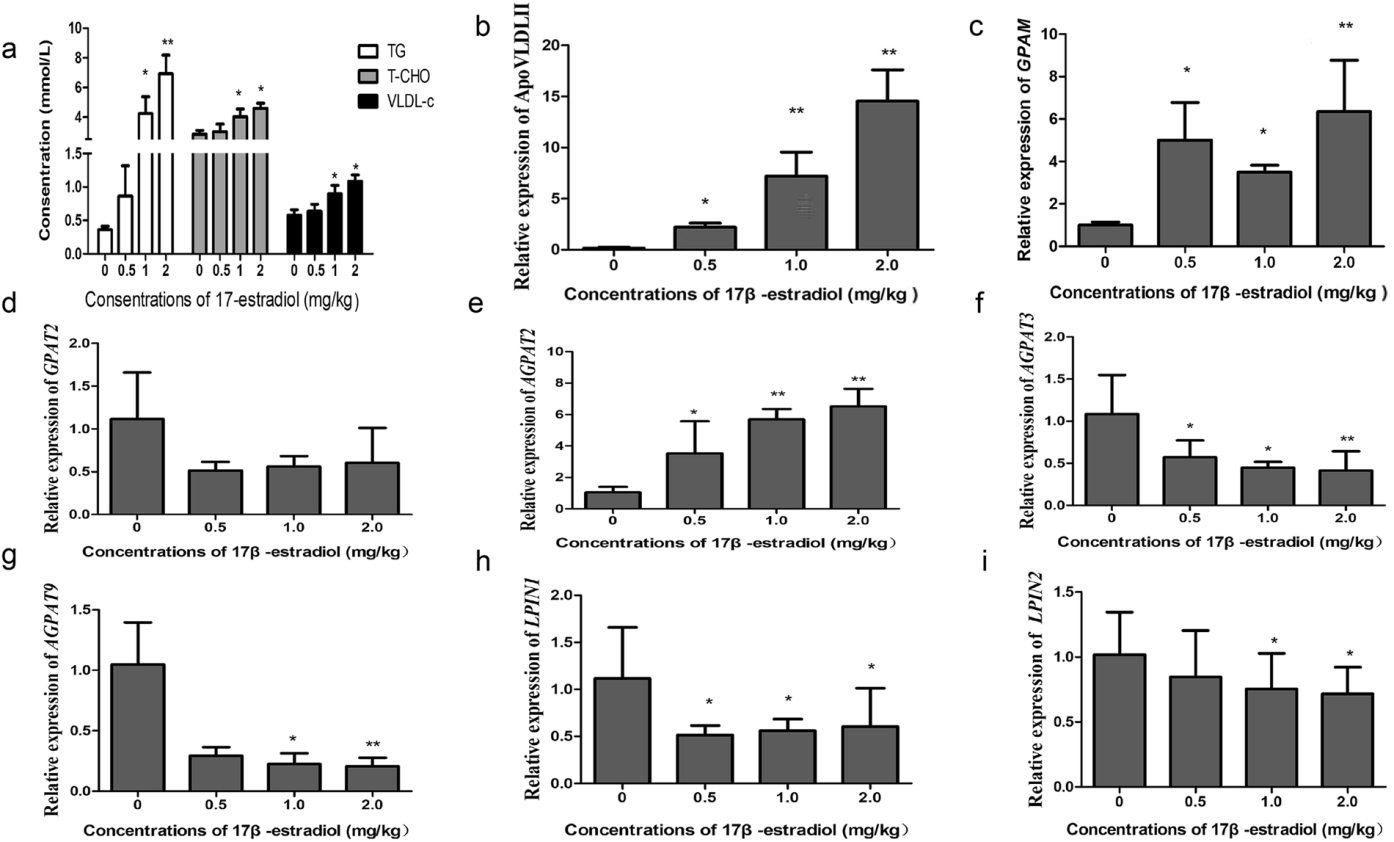
Effects of different doses of 17 β-estradiol on serum biochemical indexes and the expression of three gene families in chicken livers. (**a**) Effects of 17 β-estradiol treatment on serum biochemical indexes in chickens. (**b**–**i**) Effects of 17 β-estradiol on the mRNA expression of three gene families in chicken livers. Quantitative data are presented as the fold changes compared with gene expression in the 0 mg/kg group. For each treatment in chicken liver tissues, data were presented as the mean ± SD (n = 6); *Significant differences (*P* < 0.05), **Highly significant differences (*P* < 0.01).

Estrogen is generally considered as a major factor regulating the expression of genes related to lipid metabolism in chicken livers^39,40^. To explore the regulatory mechanisms of three gene families involved in the TG synthesis pathway, 10-week-old pullets were treated with different doses of 17β-estradiol for 12 h. After the treatment, serum biochemical parameters of the chickens were determined. Compared with control group, TG, total cholesterol (TC) and VLDL-cholesterol (VLDL-c) contents were significantly increased in chickens after receiving an injection of 17β-estradiol (1 or 2 mg/kg). The results indicated that lipid synthesis and metabolism of hens were strikingly increased with 17β-estradiol induction. *In vivo* studies on mRNA expression of *ApoVLDL II* was significantly increased in a dose-dependent manner, indicating that exogenous 17β-estradiol exerted the physiological role of estrogen in the body. Members of three gene families exhibited different responses to the treatment. In the *GPAT* family, *GPAM* expression level was remarkably increased after 12 hours of 17β-estradiol treatment and the differences in comparison with control group were either significant (*P* < 0.05) or highly significant (*P* < 0.01). However, in comparison with control group, the expression level of *GPAT2* decreased with no significant difference. In the *AGPAT* family, *AGPAT2* expression level was strikingly increased in a dose-dependent manner and the differences were either significant (*P* < 0.05) or highly significant (*P* < 0.01) compared to the control group. In contrast, in comparison with control group the expression levels of *AGPAT3* and *AGPAT9* decreased and the differences were either significant (*P* < 0.05) or highly significant (*P* < 0.01). Other members in the gene family showed no response to 17β-estradiol treatment. Both *LPIN1* and *LPIN2*, members of the *LPIN* gene family, exhibited down regulation in expression upon 17β-estradiol treatment, and the differences was significant (*P* < 0.05) (Fig. 5).

## Discussion

Triglycerides are the most abundant lipids in humans and animals, and most tissues can use their decomposition products for energy. In chickens, liver plays a vital role in lipid metabolism. In the present study, we first identified the members of three gene families in the glycerophosphate pathway on the chicken genome. Evolutionary analysis showed that members in the *AGPAT*, *GPAT* and *LPIN* families were respectively grouped into 4, 2 and 2 clusters based on their phylogenetic relationships. Orthologous genes that exist in different species were classified into the same cluster on the phylogenetic tree that were linked to a rooted clade. As reported previously, orthologous genes may be differentiated from a common ancestral gene, and the different members of the multigene family are paralogous genes that may be produced by gene replication of an ancestral gene^41^. The number of members in a multigene family is often associated with a particular living environment in different species, and some genes may be added or lost during adaptation to the living environment^41^. Moreover, the phylogenetic classification of three gene families was also supported by conserved motif and gene structure analyses. Results of motif analysis showed that the distribution of the 10 most enriched motifs were highly conserved in the same subfamily. Results of gene structure analysis showed that most closely related members in the same gene family harbored similar introns-exon structures. These particular features of conserved introns-exon structures also have been observed in plants, such as banana and Brachypodium distachyon^42,43^. Similar gene structures in each cluster indicated that gene duplication occurred in ancestral genes at ancient times, and thus descendant genes evolved into different gene structures to achieve novel functions.

Some gene expression patterns are closely related to gene functions and are conserved among species. For instance, in the *GPAT* family, *GPAM* was expressed at high level in heart, liver and kidney, whereas *GPAT2* was expressed at lower levels in liver, which was consistent with previous studies on *GPAT2* mRNA expression levels in mice^44^. In the *AGPAT* family, *AGPAT2* was highly expressed in liver and duodenum, while *AGPAT4* and *AGPAT6* were highly expressed in ovary and heart, respectively. However, *AGPAT3*, *AGPAT5* and *AGPAT9* were expressed at moderate or low levels in all the tissues. Unlike in chickens, *AGPAT2* and *AGPAT3* levels are high in white and brown fat, liver, and heart tissues in mice^45,46^. In addition, the expressions of *AGPAT2* and *AGPAT3* could be induced by disruption of the skin permeability barrier, indicating that AGPATs might play a possible role in the phospholipid or TG synthesis required for epidermal permeability barrier recovery^46^. In the *LPIN* family, *LPIN1* and *LPIN2* were broadly expressed in all tested tissues. The *LPIN1* was highly expressed in liver and ovary, and *LPIN2* was highly expressed in ovary. Interestingly, isoforms of *LPIN1* gene, *Lpin1-α* and *Lpin1-β* are highly expressed in chicken ovaries and skeletal muscles^23,36^, which may be involve in the regulation of reproduction and muscle development in chicken. In addition, the above studies showed that none of the gene family members was only expressed in a single tissue, suggesting diverse regulatory effects of member genes in these three gene families.

In chickens, liver is the most important organ for lipid metabolism. The mRNA levels of *GPAM* and *GPAT2* were lower in the early stage of growth and development (5–20 weeks old), and then *GPAM* expression level increased remarkably after sexual maturity (30–35 weeks old). Previous studies have showed that knockout of *GPAM* strikingly reduces TG synthesis and the expression of lipid metabolism-related genes in BEF cells^8^. Furthermore, the mRNA expression of *GPAT2* in rat liver did not increase after fasting, indicating that *GPAT2* contributes little to diet-induced liver TG synthesis^44^. The mRNA expression levels of AGPATs showed that *AGPAT2*, 3, 4, 5, and 6 gradually increased from 5 to 35 weeks old. However, expression of *AGPAT9* peaked in early stage of growth and development before gradually declined during pre-laying and peak-laying period. In the *LPIN* family, *LPIN1* and *LPIN2* expressed at lower levels during the early stage of growth and development (5–20 weeks old) and then increased notably during the peak laying period (30–35 weeks old). In order to synthesize nutrients to meet egg yolk formation during embryonic development, lipid synthesis increased dramatically from the early (20 weeks old) to the peak laying period (30 weeks old). Undoubtedly, the *GPAT*, *AGPAT* and *LPIN* gene families were closely related to avian lipid metabolism, and the fact that mRNA expression levels of most members of the three gene families significantly increased after sexual maturity suggests that these genes play an irreplaceable role in lipid metabolism. Further analyses need to be performed to further elucidate their specific mechanisms in liver.

Estrogen is critical to lipid synthesis during the peak laying period of female hens. Previous researches have indicated that the estrogen concentrations in the plasma of female chickens reach their highest levels before the first egg was produced, and then decreased gradually^47^. In addition, estrogen targets liver, and was generally considered as a major factor that regulates the expression of genes related to lipid metabolism in chicken livers^48,49^. In this study, the effect of estrogen on the induction of the gene expression was investigated. Analyses of serum biochemical indexes showed that the concentrations of TG, TC and VLDL-c were increased significantly after 17β-estradiol treatment, suggesting that the rate of lipid metabolism in hens was remarkably enhanced. After 17β-estradiol treatment, mRNA expression levels of *GPAM* and *AGPAT2* were significantly increased, while expression of *AGPAT3*, *AGPAT9*, *LPIN1* and *LPIN2* were significantly decreased. Other genes tested have shown no expression regulations. It implied that the elevation of gene expression levels during peak-laying period was not simply regulated by estrogen alone. In fact, with the arrival of the egg production peak, other hormones in addition to estrogen such as luteinizing hormone (LH) and follicle-stimulating hormone (FSH) also altered their activities. Therefore, the changes of expression levels of the three gene family members during peak-laying period could be controlled by a combination of multi-factors. However, the specific mechanisms underlying the phenomenon need to be studied further.

## Conclusion

In animal production, lipid metabolism is closely related to animal growth and development, feed conversion ratio, and meat quality. Liver is the main organ of chicken lipid metabolism and accounts for approximately 90% of *de novo* synthesis of fatty acids in chicken. Glycerophosphate synthesis pathway play a key role in TG synthesis in chicken. Analysis of the evolution and expression characteristics of gene family members related to this pathway revealed that the orthologous genes exist in different species were classified into the same cluster in the phylogenetic tree and were linked to a rooted clade. The 10 most enriched motifs were highly conserved in the same subfamily and most closely related members in the same gene family harbored similar introns-exon structures. These genes were ubiquitously expressed in a variety of tissues, and their expression levels changed dynamically in the liver during different growth and development periods. After 17β-estradiol treatment for 12 h, the mRNA expression levels of *GPAM* and *AGPAT2* were markedly increased, *AGPAT3*, *AGPAT9*, *LPIN1* and *LPIN2* were remarkably decreased, whereas others had no change. These findings lay the foundation for further analysis of the mechanism of lipid metabolism in chicken liver.

## Materials and Methods

### Identification of members of the *GPAT*, *AGPAT and LPIN* in the chicken genome that involved in the glycerophosphate pathway

The nucleotide and protein sequences of the *GPAT*, *AGPAT* and *LPIN* gene family members in humans and mice were used as queries to search for members of the three gene families against nucleotide and protein sequence databases in chickens by NCBI BLASTN and BLASTP tools (https://www.ncbi.nlm.nih.gov/) and BLAST/BLAT search at the Ensembl genome browser (http://asia.ensembl.org). For convenience, the longest transcript was selected when genes had more than one transcript.

### Gene structure and phylogenetic analyses of the *GPAT*, *AGPAT and LPIN* in chicken

All amino acid sequences of members of the three gene families in chicken and other species, including mammals (human, mouse and cow), avians (chicken and falcon), amphibians (tropical clawed frog) and fish (zebrafish), were retrieved from the NCBI protein database. The phylogenetic tree was constructed using MEGA 6.0 software by the neighbor-joining (NJ) method, based on the alignment of protein sequences between other species of three families by Clustal W. The reliability of the tree was assessed using 1000 bootstrap replicates. The numbers at each clade represent bootstrap support values given as percentages. To understand the biological function of the *GPAT*, *AGPAT* and *LPIN* gene families in chickens, their conserved motifs were analyzed by MEME software (http://meme-suite.org/). BioMart (http://asia.ensembl.org/biomart/martview/) was used to obtain exon-intron structure information for all gene family members, and GSDS (http://gsds.cbi.pku.edu.cn/) was used to map gene exon-intron structure. The heat maps were drawn using IBS v.1.0. software (http://ibs.biocuckoo.org/).

### Animals, treatment and sampling

All animal experiments were conducted according to the Institutional Animal Care and Use Committee guidelines, following protocols approved by the Henan Agricultural University (Permit Number: 11-0085). An indigenous Chinese breed, the Lushi blue-shell-egg chicken, was used for this study.

To investigate the expression profiles of the three gene families, tissues including heart, liver, spleen, lung, kidney, pancreas, duodenum and ovary were collected at 10 and 30 weeks old (n = 6 for each group). In addition, 5, 15, 20, 30 and 35 weeks old livers were collected to study their spatial and temporal expression patterns (n = 6 for each group). All of the samples were snap frozen in liquid nitrogen and then stored at −80 °C for further use.

To investigate the effect of estrogen on the expression regulation of genes of the three families, an *in vivo* study was carried out as described previously^39^. Briefly, 40 healthy Lushi blue-shell-egg chickens at 10 weeks of age were randomly divided into a control group and three treatment groups. Birds in the three treatment groups were subcutaneously injected with 0.5, 1.0, or 2.0 mg/kg body weight 17-β estradiol (Sigma, St. Louis, MO, USA) (dissolved in olive oil). Birds in the control group were subcutaneously injected with an equal amount of the solvent. All the birds were euthanized and their livers were collected after 12 h of treatment. The sample collection procedure is the same as mentioned above.

### RNA extraction and reverse transcription

TRIzol® reagents (TaKaRa, Kyoto, Japan) were used to extract the total RNA from difference tissues and each of the RNA samples was treated with DNase I to remove gDNA. The RNA purity and concentration were assessed by using NanoDrop2000 spectrophotometer (Termo Scientific, Wilmington, DE, USA) and RNA integrity was analyzed by using denatured agarose gel electrophoresis. The RNA samples with OD260/280 ratios above 1.8 and the 28S and 18S bands with brightness in denatured agarose gel was selected for further analysis. The RNA was reverse-transcribed into cDNA by using a Prime Script^TM^ RT Reagent Kit (TaKaRa, Kyoto, Japan) according to the manufacturer’s protocol. The cDNA was then stored at −20 °C until use.

### Quantification of serum biochemical indicators

The contents of serum TG, TC, LDL-c (low-density lipoprotein cholesterol) and HDL-c (high-density lipoprotein cholesterol) were determined by automatic blood analyzer (Hitachi 7100, Japan). The VLDL-c concentration was calculated according to the Friedewald formula, VLDL-c = TG-HDL-c-LDL-c.

### Quantitative real-time PCR (qPCR)

To investigate the expression changes of members of the three gene families in chickens, SYBR green-based real-time PCR analysis was performed using a LightCycler96 Real-Time PCR system (Roche Applied Science, Switzerland). The primers were designed using the Pick-primers tool of NCBI and synthesized by Sangon Biotech (Shanghai, China) (see Supplementary Table S2). The primers are located at the common site of coding sequences among all alternative splice variants and spanned at least one exon-intron junction. The total reaction volume was 10 µl, which contained 5 µl of 2 × SYBR® Premix Ex Taq™ II (Takara, Kyoto, Japan), 0.5 µL each of forward and reverse primers (10 µM), 1 µl of cDNA and 3 µl of RNase-free water. All reactions were executed as follows: predenaturing were performed at 95 °C for 5 min, followed by 40 cycles of 30 s of denaturing at 95 °C, 30 s of annealing at 60 °C and 30 s of extension at 72 °C, then a further 10 min of extension occurred at 4 °C. The graphics were drawn by GraphPad Prism 5 (GraphPad, San Diego, CA, USA).

### Statistical analysis

The relative gene mRNA expression levels were assessed using the 2^−△△CT^ method with β-actin as the reference gene. Statistical analysis was performed by one-way ANOVA followed by Dunnett’s test using SPSS version 20.0 software, and the data are presented as the mean ± standard deviation (SD). *P* < 0.05 was considered statistically significant and *P* < 0.01 was considered highly statistically significant.

## Supplementary information

Evolution, dynamic expression changes and regulatory characteristics of gene families involved in the glycerophosphate pathway of triglyceride synthesis in chicken (Gallus gallus)
