# Supplementary material for: Evolution, dynamic expression changes and regulatory characteristics of gene families involved in the glycerophosphate pathway of triglyceride synthesis in chicken (Gallus gallus)
Source: Sci Rep. 2019 Sep 4;9:12735. doi: 10.1038/s41598-019-48893-9 (PMC6726641; doi:10.1038/s41598-019-48893-9)
Supplement: Supplementary file 1 — Evolution, dynamic expression changes and regulatory characteristics of gene families involved in the glycerophosphate pathway of triglyceride synthesis in chicken (Gallus gallus) [file 41598_2019_48893_MOESM1_ESM.pdf]

Evolution, dynamic expression changes and regulatory characteristics of gene families involved in the glycerophosphate pathway of triglyceride synthesis in chicken (*Gallus gallus*)

Liyu Yang[1], Ziming Liu[1], Kepeng Ou[2], Taian Wang[1], Zhuanjian Li[1,3,4], Yadong Tian[1,3,4], Yanbin Wang[1,3,4], Xiangtao Kang[1,3,4], Hong Li[1,3,4]*, Xiaojun Liu[1,3,4]*

[1]College of Animal Science and Veterinary Medicine, Henan Agricultural University, Zhengzhou 450002, China

[2]Academic Unit of Ophthalmology, Bristol Medical School, University of Bristol, Bristol BS8 1TD, UK

[3]Henan Innovative Engineering Research Center of Poultry Germplasm Resource, Zhengzhou 450002, China

[4]International Joint Research Laboratory for Poultry Breeding of Henan, Zhengzhou 450002, China

*Corresponding authors.

Xiaojun Liu: xjliu2008@hotmail.com

Hong Li: lihong19871202@163.com

Supplementary informations

Table S1.a. The species names and NCBI accession numbers of GPAT family for the phylogenetic tree construction

Gene	Species	NCBI accession numbers
GPAT2	Mm, Mus musculus (house mouse)	NP_001074558.2
	Dr, Danio rerio (zebrafish)	NP_001082849.1
	Hs, Homo sapiens (human)	NP_001082849.1
	Xt, (Xenopus(Silurana) tropicalis (western clawed frog)	XP_017947975.1
	Gg, Gallus gallus (chicken)	XP_015128446.1
	Bt, Bos taurus (cattle)	NP_001012282.1
	Fp, Falco peregrinus (peregrine falcons)	NP_001179563.1
GPAM	Hs, Homo sapiens (human)	NP_055461.1
	Bt, Bos taurus (cattle)	NP_001012282.1
	Dr, Danio rerio (zebrafish)	XP_009305069.1
	Xt, (Xenopus(Silurana) tropicalis (western clawed frog)	NP_001072564.1
	Gg, Gallus gallus (chicken)	NP_001072564.1
	Mm, Mus musculus (house mouse)	NP_001343214.1
	Fp, Falco peregrinus (peregrine falcons)	XP_005239302.1

Table S1.b. The species names and NCBI accession numbers of AGPAT family for the phylogenetic tree construction

Gene	Species	NCBI accession numbers
AGPAT1	Hs,Homo sapiens (human)	NP_006402.1
	Bt, Bos taurus (cattle)	NP_803484.1
	Xt, (Xenopus (Silurana) tropicalis (western clawed frog)	NP_001090802.1
	Gg, Gallus gallus (chicken)	XP_001233846.2
	Mm, Mus musculus (house mouse)	NP_001156851.1
AGPAT2	Hs, Homo sapiens (human)	NP_001012745.1
	Bt, Bos taurus (cattle)	NP_001073733.1
	Xt, (Xenopus (Silurana) tropicalis (western clawed frog),	NP_001123741.1
	Gg, Gallus gallus (chicken),	XP_001235300.2
	Mm, Mus musculus (house mouse),	NP_080488.1
	Dr, Danio rerio (zebrafish),	NP_001071200.1
	Fp, Falco peregrinus (peregrine falcons)	XP_005231387.1
AGPAT3	Hs,Homo sapiens (human)	NP_001032642.1
	Bt, Bos taurus (cattle)	NP_001033135.1
	Xt, Xenopus (Silurana) tropicalis (western clawed frog)	NP_001008119.1
	Gg, Gallus gallus (chicken)	XP_004934657.1
	Mm, Mus musculus (house mouse),	NP_443747.2
	Dr, Danio rerio (zebrafish)	NP_998590.1
	FP, Falco peregrinus (peregrine falcons)	XP_005234551.1
AGPAT4	Hs, Homo sapiens (human)	XP_016866548.1
	Bt, Bos taurus (cattle)	NP_001015537.1
	Xt, Xenopus (Silurana) tropicalis (western clawed frog)	NP_001011265.1
	Gg, Gallus gallus (chicken)	XP_419616.2
	Mm, Mus musculus (house mouse),	NP_080920.2
	Dr, Danio rerio (zebrafish)	NP_001035339.2
	Fp, Falco peregrinus (peregrine falcons)	XP_005230336.1
AGPAT5	Hs, Homo sapiens (human)	NP_060831.2
	Bt, Bos taurus (cattle)	XP_005225981.1
	Xt, Xenopus (Silurana) tropicalis (western clawed frog)	NP_001015964.1
	Gg, Gallus gallus (chicken)	XP_419916.1
	Mm, Mus musculus (house mouse)	NP_081068.1
	Dr, Danio rerio (zebrafish)	NP_001070213.2
	Fg,Falco peregrinus (peregrine falcons)	XP_005238188.1
AGPAT6	Bt, Bos taurus (cattle)	NP_001077138.1
	Xt, Xenopus (Silurana) tropicalis, (western clawed frog)	NP_001123822.1
	Gg, Gallus gallus (chicken)	XP_015152891.1
	Mm, Mus musculus (house mouse),	NP_061213.2
	Dr, Danio rerio (zebrafish)	NP_001035339.2
	Fp, Falco peregrinus (peregrine falcons)	NP_001123822.1
AGPAT9	Bt, Bos taurus (cattle)	NP_001179443.1
	Xt, Xenopus (Silurana) tropicalis (western clawed frog)	NP_001087492.1
	Gg, Gallus gallus (chicken)	NP_001026316.1
	Mm, Mus musculus (house mouse),	NP_766303.1
	Dr, Danio rerio (zebrafish)	NP_001002685.1

Table S1.c. The species names and NCBI accession numbers of LPIN family for the phylogenetic tree construction

Gene	Species	NCBI accession numbers
LPIN1	Hs, Homo sapiens (human)	NP_001336136.1
	Dr, Danio rerio (Zebrafish)	NP_001037818.1
	Gg, Gallus gallus (chicken)	XP_015131575.1
	Mm, Mus musculus (house mouse),	NP_001123884.1
	Xt, Xenopus (Silurana) tropicalis (western clawed frog)	XP_005234551.1
	Fp, Falco peregrinus (peregrine falcons)	XP_005238227.1
	Bt, Bos taurus (cattle)	NP_001193085.2
LPIN2	Gg, Gallus gallus (chicken)	NP_001006386.2
	Hs, Homo sapiens (human)	NP_055461.1
	Mm, Mus musculus (house mouse),	NP_001158357.1
	Dr, Danio rerio (zebrafish)	XP_005163511.1
	Xt, Xenopus (Silurana) tropicalis (western clawed frog)	NP_001072517.1
	Bt, Bos taurus (cattle)	XP_010817245.1
	Fp, Falco peregrinus (peregrine falcons)	XP_013153869.1

Table S2 List of the genes and primers used for qRT-PCR validation

Gene name	Forward primer(5'-3')	Reward primer(5'-3')	PCR product
ApoVLDLII	CAATGAAACGGCTAGACTCA	AACACCGACTTTTCTTCCAA	108
β-actin	GAGAGAAGATGACACAGATC	GTCCATCACAATACCAGTGG	116
GPAM	TCCATCGAGACCTAATGATAC	TAGACATCACAGCACAGGAC	182
GPAT2	GTTTTCCTCTCCACCCACAAG	CTCAATGCCTGTTCCACTCTC	155
AGPAT2	CACCGTCAAGAACATGAGGA	ACCTCCATCAGCCCCATCAT	165
AGPAT3	AAGACCCAGTTCATTGTTC	ACCACCATTCCAGCAGCATT	180
AGPAT4	CAGTGGCATAACCTGGAGCA	CAGCACACCCAGCTGTTTTC	246
AGPAT5	CTGGCACCCATCTCAGTGTT	CAGGTGAGAGGACGCACAT	148
AGPAT6	CGTTCACTGGCATCAGCTTG	CGTCAATGGGGGATGTGTGA	213
AGPAT9	GACCGCCATCTAGTGACAAA	GAACGCATCTCCAAACTGA	189
LPIN1	TAATGAGAGACAAGATGCCC	ATCTTTTATTCTGTTTGCCAT	165
LPIN2	CCACATCTCCAATACCCACT	AGTCTCTGTTTCCATAGCAT	122
